# Exploring Vocabularies of Motive to Optimise Patient Engagement for Drug Development

**DOI:** 10.1111/1467-9566.70274

**Published:** 2026-10-13

**Authors:** Julia Frost, Widening Engagement Patient Advisory Group, Jessica Mandizha, Chantal van den Dungen, Catherine Pope

**Affiliations:** ^1^ Health and Community Sciences University of Exeter Medical School University of Exeter Exeter UK; ^2^ Royal Devon University Healthcare NHS Foundation Trust, Royal Devon and Exeter Hospital (Wonford) Exeter UK; ^3^ ABFFP Association Belge Francophone contre la Fibrose Pulmonaire Rebecq Belgium; ^4^ Nuffield Department of Primary Care Health Sciences Oxford UK

**Keywords:** drug development, ethnography, patient engagement, return on engagement, return on investment, vocabularies of motive

## Abstract

Patient engagement in the drug development life cycle is becoming normalised, and the return on investment is being quantified. We sought to understand what motivates biotechnology organisations to undertake patient engagement throughout the drug development life cycle, to enable us to generate theoretically and empirically grounded knowledge to optimise patient engagement. This paper draws upon ethnographic research including 200 hours of informal observation, 50 hours of formal observation, 10 interviews with biotechnology staff and documentary analysis. With patient input throughout this research, we identified a multitude of motives for patient engagement, including regulatory requirements, corporate social responsibility and return on investment. Less evident was the translation of those motives into a framework that could optimise patient engagement over time and at scale. This was due to the dynamic nature of the drug pipeline, the limited critical space in which patient engagement can occur and misconceptions about patient engagement mechanisms, incentives and metrics. Exploring existing vocabularies of motive within an industry‐led initiative identified organisational practice in both drug development processes and wider industry practices that have the potential to inhibit meaningful and sustainable engagement, and thus limit translation and optimisation of discovery‐to‐prescription translation for a wider group of patients.

## 1 Introduction

This paper mobilises the neglected concept of motive (Campbell 1991), specifically the elaboration of Weber's ideas by C Wright Mills in his paper *Situated Actions and Vocabularies of Motive* (Mills 1940), to understand patient engagement in contemporary drug development. We do so to explain what enables meaningful patient engagement in the development of drugs but also to offer vocabularies of motive as a conceptual lens through which to assess whose interest an organisation’s patient engagement strategy may or may not serve.

### 1.1 Patient Engagement for Drug Development

Over 90% of clinical drug development trials fail. Many failures are due to clinical inefficacy, unmanageable toxicity, inadequate drug‐like properties and lack of commercial need (Sun et al. 2022). To reduce the probability of late‐stage failure and address challenges associated with poor recruitment and retention in clinical trials, patient engagement has been proposed as a potential solution (Zvonareva 2023). Patient engagement has been mandated by regulatory authorities (e.g., the European Medicines Agency and US Food and Drug Administration) (Food and Drug Administration 2022; European Medicines Agency 2025) and championed by industry and stakeholder collaborations (e.g., Patient Focused Medicines Development—PFMD) (Egher and Zvonareva 2023). Trials that have involved patients in the design and execution have been lauded for reducing recruitment times (Economist Intelligence Unit 2018) and producing a 500‐fold increase in return on investment (Levitan et al. 2018).

There are over 60 frameworks for patient engagement in research and a variety of tools for incorporating patients in the drug development process (Nicholls and Campbell 2024). Pharmaceutical companies have developed toolkits such as the TransCelerate Patient Protocol Engagement Toolkit (P‐PET) (https://www.transceleratebiopharmainc.com/ppet/planning‐for‐patient‐engagement/) (TransCelerate Biopharma Toolkits Core Team et al. 2020) and Patient Focused Medicine Developments' Engagement Management Suite (https://pemsuite.org/), which contain user guides, resources and templates for standardising and quantifying the facilitation of patient engagement activities. In industry‐led frameworks, ‘patient intelligence’ refers to the strategic process of evidence gathering (increasingly with the assistance of AI), whereas the ‘patient voice’ refers to the product of gathering lived experience (which may also include personal health data) to be integrated into decision‐making. However, critics contend that much patient engagement by industry currently reinforces existing inequalities, as industry works with ‘representatives’ who are easiest to engage with on activities restricted to those with limited influence (Sayani and Maybee 2025). Empirical studies have identified that biotechnology staff are sceptical about the value of working with patients (Morretti and Sacchetti 2024) and downplay the extent to which patient organisations (POs) are forced to compete to be engaged by industry (Auwal et al. 2025). Furthermore, patient engagement remains transactional, as it is one of many business considerations, alongside timelines, cost and manufacturability (Biondi et al. 2024). In contrast, taking a translational justice approach, defined as procedural and outcomes‐based attention to how medical technologies move from the lab to the patient in a way that equitably addresses the values and needs of those most morally impacted, would require a wider group of patients to be engaged earlier in the drug development process, sustained beyond the development of one new medicine or therapy and rewarded fairly for their contribution (Allyse et al. 2025). As such, these practices are at odds with the market‐led pharmaceutical business model, and examples of truly equity‐focused practice, as in the creation of a pharmaceutical commons, are rare (Geiger and Kleinhout‐Vliek 2026).

### 1.2 Vocabularies of Motive

For C. Wright Mills, vocabularies of motive (the ‘names given for consequential actions, and surrogates for action leading to consequences’) are important because they articulate the accepted justifications for acts and programmes which can co‐opt allies into strategies of actions and provide explanations for those social actions (Mills 1940, 905). Mills suggests that ‘we may analyse, index, and gauge’ which types of vocabularies of motive can provide mechanisms of action that constitute a highly specified ‘programme’ of anticipated consequences in a given context (Mills 1940, 911). However, where Weber (1947) and Burke (1945) viewed motive as grounds for an individual's conduct, conversely Foote (1951) saw action as dictating the account rather than guiding it, whereas Scott and Lyman (1968, 47) defined ‘socially approved vocabularies’ (or excuses) as mitigating behaviour or ‘relieving responsibility when conduct is questioned’. Thus, vocabularies of motive provide a framework for the exploration of mechanisms of organisational practice; for example, by identifying where vocabularies might be used to disrupt the legitimacy of the status quo (Sykes and Matza 1957) or where disclaimers are employed to further legitimise existing claims made by managers (Hewitt and Stokes 1975).

Despite the development and application of Mills' notion of vocabularies of motive, Bruce and Wallis (1983) considered this approach, and discourse analysis more generally, as merely *post‐hoc rationalisation*. However, this perspective ignores Gerth and Mills' (1953) assertion that motives are used strategically and purposefully to influence, control and persuade others to further one's conduct, particularly in business settings (e.g., they can be future‐oriented). Subsequently, Suddaby and Greenwood (2005) have suggested that institutional vocabularies both articulate a particular corporate reality and provide a means for interpreting those vocabularies. They propose that language has the potential to shift organisational logics, which then alter the legitimacy of organisational forms (Suddaby and Greenwood 2005). Similarly, in their exploration of the notion of ‘lived experience’, McIntosh and Wright (2019) have proposed that conducting ethnography while attuned to vocabularies of motive enables the identification of actors who never talk quite the same language. Thus, where discourse analysis identifies the structuring of language, vocabularies of motive foreground why actors chose specific justifications for their actions and negotiations with other actors in a given context, specifically contexts undergoing historical change or conflict.

### 1.3 Context of the Project

As part of a knowledge transfer partnership funded by a UK Research and Innovation/Medical Research Council (UKRI/MRC) Innovation Scholars Secondment, JF was seconded to a biotechnology company (hereafter ‘Biotech’), as an embedded researcher (Marshall et al. 2014) over a 3‐year period. Commencing a six‐year transformation project, Biotech planned to embed patient engagement in drug development and had established relationships with both disease‐specific patient organisations (POs) and umbrella POs. To identify how a wider demographic of patients could be enabled to participate in both clinical trials and trial design, JF worked with another medical sociologist (CP), clinical staff (including JM), a patient support group (patient advisory group), a local patient organisation (Exeter Patients in Collaboration for Pulmonary Fibrosis [EPIC PF]) and expert patient organisations (Action for Pulmonary Fibrosis; Association Belge Francophone contre la Fibrose Pulmonaire; European Pulmonary Fibrosis Federation) which subsequently constituted the Steering Group for the project along with Biotech.

Following best practice guidance from the MRC, we convened a patient advisory group (PAG), which we met with regularly to inform the scope and content of the research. The PAG were active in developing the scope and content of the research proposal; they suggested topics for exploration, were actively engaged in the analytical process—providing validation of the researchers' interpretations, and they produced a set of recommendations for Biotech. The PAG consisted of six people with idiopathic pulmonary fibrosis (IPF) or who care for someone with IPF. The research was also regularly discussed with a Steering Group (SG) detailed above. Members of the PAG and SG provided insights into their own motives and experiences for participating, or not, in clinical trials and clinical trial design, and similarly provided their interpretations of study findings as the research developed.

This paper explores the enactment of an industry‐led patient engagement strategy for drug development. It contributes to the study of both the implementation of market‐focused patient engagement in the pharmaceutical landscape and critical studies of patient engagement. It does so by drawing on Mills' conceptualisation of vocabularies of motive to recalibrate our understanding of how discourse reflects the meaning of ‘engagement’ to offer a more critical perspective on how such collaborations are framed and circumscribed.

## 2 Methods

### 2.1 Sampling and Recruitment

The sampling frame for fieldwork was premised on the linear development of patient engagement processes (e.g., policy development vis‐à‐vis internal meetings with different functions) and their cyclical enactment in engaging different patient representatives (e.g., external encounters with contract research organisations, CROs, and patient organisations, POs), as new clinical trials were planned (Tavory and Timmermans 2014). The sampling frame for interviews was any member of staff currently employed by Biotech or its subsidiaries, who was involved in patient engagement explicitly or who interacted with staff engaged in patient engagement (Malterud et al. 2016).

For fieldwork, as stipulated in Biotech company policy, a privacy notice was shown prior to all meetings that the industry host identified as acceptable for formal observations, with verbal consent specified as the preferred method of consent by the host organisation (Crowhurst and Kennedy‐Macfoy 2013). Thus, some formal observations were limited to parts of site visits or meetings, whereas field notes were permitted to capture informal observations. Staff were given the opportunity to withdraw from being observed, without needing to provide a reason. For interviews, staff who matched the sampling frame were identified by the industry host (Staff Member 1, hereafter S1). Potential participants were contacted by S1, and JF provided them with a participant information sheet about the research. If they agreed to be interviewed, they then provided written informed consent. JF arranged to interview the participant at a convenient date and location (e.g., face to face or via MS Team/Zoom). Throughout the process, participants were reminded of their right to withdraw their consent at any time, without needing to provide a reason.

JF had access to all company documents pertaining to patient engagement during the secondment and undertook statutory industry training to access those materials securely, but due to their proprietary content, consent was not provided for them to be shared. Thus, contractual arrangements with Biotech controlled and restricted access to different aspects of the development of the patient engagement strategy.

### 2.2 Data Collection

Field notes were made using pen and paper or an encrypted laptop as appropriate. Documents about the patient engagement transformation project (e.g., underpinning sources or documents said to be co‐created with patient representatives) were collected to explore current and planned situational actions pertaining to patient engagement. Because of their proprietary content, only publicly available sources within them, such as PFMD, can be disclosed. Semi‐structured interviews with staff and formal observations of meetings were planned to capture the roll‐out of the patient engagement transformation project and coincided with the discontinuation of the drug development pipeline for IPF (Rapport and Braithwaite 2020). The focus of the interview and observation guides, co‐developed with PAG members, was staff orientations to patient engagement, aspects of the organisation's patient engagement transformation project, and perspectives on the value and possible remit of patient engagement more generally. Interviews were conducted face to face or remotely, as the participant preferred. The interview and topic guides were tailored to the context, for example, depending on a person’s role within a function and where the observation occurred in the transformation project. All interviews were transcribed verbatim by a GDPR‐compliant transcriber and, with observational field notes and industry documents, anonymised and managed in MAXQDA software (VERBI Software 2021). As part of the wider ethnography, interviews with people who have IPF and clinical staff who recruit them to clinical trials were also undertaken to better understand patient nee*ds* (Frost et al. 2025a; Frost et al. 2025b).

### 2.3 Data Analysis

Data were analysed using analytical abduction, which tested findings from an earlier literature review (e.g., the use of existing philosophical and methodological frameworks for engaging patients, appropriate methods of data collection and analysis to gauge ‘engagement’ rather than ‘satisfaction’ and dissemination of patient‐facing materials) (Frost et al. 2023) and identified the perspectives and practices of those involved in Biotech's patient engagement strategy and practices, and the wider drug development context (Hammersley and Atkinson 2019). The unique perspective, afforded by researching in situ and discussing inferences among a ‘community of inquiry’, populated by patients, clinicians and industry participants (our PAF and SG co‐authors), enabled the production of a reasoned argument based on empirical evidence (Tavory and Timmermans 2014) to account for ‘present, future or past programs or acts’ (Mills 1940, 907). Data were anonymised and participants given pseudonyms (*S* designates ‘staff member’ and *P* ‘patient representative’).

From June 2021, JF undertook informal observations (over 200 hours) and documentary analysis. Ethical approval was delayed due to COVID‐19; however, from April 2022 (when ethical approval was granted) until July 2024, JF continued to attend meetings and collect informal observations and documents. Formal observations of team meetings and meetings with internal and external stakeholders (*n* = 12, totalling 50 hours) and semi‐structured interviews were conducted with staff (*n* = 10) (Table 1).

**TABLE 1:** Formal observation and interview sample.

Formal observations (*n* = 12)		Interviews (*n* = 10 people)
Face to face (*n* = 3)	Remote (*n* = 9)	
Patient engagement (PE) functional team meeting (2) Patient voice* session at patient day (1)	Patient Steering Group (1) Patient Steering Group meeting (1) Patient engagement roadshow (1) Patient engagement team meeting (1) Engagement team meeting (2) Engagement meeting with patient organisation (2) Sustainability meeting (1)	Clinical study owner (1) Communications (1) Contract research organisation lead (1) Clinical sub‐team lead (1) Medical affairs lead (1) Patient organisation representative (1) Research programme lead (1) Engagement leads (3)

*Patient voice is a broader and more holistic term than patient experience data.

## 3 Results

Informed by the concept of vocabularies of motive, our analysis identified a range of motives for patient engagement, including regulatory requirements, corporate social responsibility and return on investment. These are foregrounded in the industry‐developed tools as socially acceptable and learnt justifications—which Gerth and Mills (1953) refer to as *premium* motives. Less evident was the translation of those motives into a framework that could optimise patient engagement over time and at scale. This was due to the dynamic nature of the drug pipeline (notably the discontinuation of a drug in development), the limited critical space in which patient engagement could occur and misconceptions about the nature of patient engagement. These latter motives feature less prominently in the standardising frameworks, and we view these as *tabooed* motives (Gerth and Mills 1953) because discussion of them is repressed, restricted or contested.

### 3.1 Premium Motives

Unsurprisingly, some of the motives identified in this analysis mirror normative justifications for patient engagement in drug development presented in the industry‐developed toolkits and guidance documents—for example, legal and financial motives. As patient engagement was a relatively new function in this Biotech, these externally produced motives were used as directives by the Patient Engagement Functional Team to incentivise buy‐in from colleagues and positioned externally (e.g., on Biotech's webpages) to showcase the organisational commitment to developing new medicines for conditions with high unmet need, as well as a wider and sustained contribution to society.

#### 3.1.1 Regulatory Requirements

Drug development is tightly controlled to ensure the safety, efficacy and quality of the drugs that are tested and become available to patients (Chisholm and Critchley 2023). Patient engagement practices were often discussed in terms of whether they were compliant with relevant laws and regulations (e.g., European Medicines Agency 2025), with a regulatory imperative seen as driving change:

the [patient engagement] process, not only in [Biotech] but in the whole industry is a trend and the new way of working, also the regulators that are asking and are putting the patients [in] their place is something completely new for us

(S2 interview 07/22)

This perception informed discussions concerning which aspects of engagement were liable to be reported to regulators:

S3: We have a legal requirement to document where recommendations have not been included or addressed. That is liable at audit.

(Team meeting 09/22)

Once a pilot patient engagement exercise had been conducted, the narrative focused upon the number of recommendations that had been accepted and feeding back to a patient organisation which recommendations had not been accepted, rather than how the accepted recommendations had been operationalised in practice:

P1: the [patient advisors] who took part in the first part of the project provided a total of 28 recommendations, and you can see that from that 28, 24 were implemented. So, the patient voice does matter, and by ensuring that patients take part, from the very early stages, to completion, we can actually make a real, real difference

(Patient day 11/23)

In this meeting with patient representatives, the emphasis was on the information that was notifiable to regulators, rather than how patient insights might enable recruitment. As such, there was no documented response from the clinical team as to how the concerns raised by patient representatives were to be addressed in the operational set‐up of a trial. Regulatory uncertainty around how to do patient engagement (with no definitive guidance available at that time from the European Medicines Agency (EMA) or the International Council for Harmonisation of Technical Requirements for Pharmaceuticals for Human Use (ICH 2021)) meant that recourse (Stephens et al. 2013) was made to the regulations that were clearly defined and understood (such as the European Union 2014). An oft‐cited example was a delay to a trial amendment because a submission had not included ‘the patient voice’, and discussions around whether this was notifiable to the regulator or not (team meeting 09/23). In this meeting with patient representatives, the emphasis was on the information that was notifiable, rather than how specific patient insights might enable recruitment.

#### 3.1.2 Perceptions of Patient Engagement

In the development phase of Biotech's patient engagement transformation process, Biotech's Patient Engagement Functional Team drew on the European Patients Academy on Therapeutic Innovation (EUPATI) website and PFMD frameworks website to inform their patient engagement transformation project. Three workshops to develop Biotech's Patient Engagement Framework were facilitated by internal and external consultants, with the objective ‘to systematically embed patient engagement across the medicines lifecycle’. The workshops identified the scope of the patient engagement initiative (to build a patient engagement framework and to implement patient engagement), with the first two workshops exploring how to achieve buy‐in among ‘colleagues [who] do not fully understand the benefits of patient engagement and might perceive this as something that might slow down planning’ (Workshop 1 06/21) and the ‘need to demonstrate impact for the organisation’ (Workshop 2 06/21). Thus, the motives for the internal change process were initially defined as company‐facing, and ‘the need to provide tools and resources’ to operationalise the objective was identified, and a set of internal resources was drafted. In the third workshop, the orientation was more towards the desire to ‘develop a sustainable framework’ to track impact, and to ‘establish endpoints that are important to patients to ensure that we bring drugs to market that people need’ and a need for projects that ‘showcase patient engagement’ (Workshop 3 07/21). Subsequently, a document was circulated that defined the objective as ‘to understand better the patients’ unmet needs, perspectives and preferences for future treatments' (cross‐team meeting 10/23); thus, the initial motives for influencing internal partners had been recast as meeting the unmet needs of patients. By 2024, as the drug development pipeline stalled and challenges in embedding patient engagement became evident, the ambition once again became business‐facing, with a framework designed ‘to support systemic patient engagement across the medicine lifecycle, and to embed patient engagement thinking across the organisation’ (Patient Engagement Functional Team document 06/23).

#### 3.1.3 Corporate Social Responsibility

Corporate social responsibility (CSR), defined as a management practice by which pharmaceutical companies integrate social and/or environmental concerns into their business operations, stakeholder interactions and public reporting (Wong et al. 2025), was viewed as an additional external ‘lever’ for ‘crowdsourcing’ internal support for the patient engagement transformation project, as the Patient Engagement Functional Team identified that patients were currently ‘missing’ from the CSR model (cross‐team meeting 09/22). Subsequently, this narrative was extended to whether inclusivity and diversity could also be incorporated into the CSR model (cross‐functional meeting 5/23), in line with the EU Corporate Sustainability Reporting Directive (2014). With the adoption of patient engagement into the CSR strategy, came discussions of how best to measure impact:

S1 introduced slides depicting potential key performance indicators, informed by PFMDs metric selector, for patient engagement, including ‘% of studies with patient centric endpoint design, % of Phase 2b‐4 studies including patient voice, % of patient recommendations actually implemented, and a Study participant experience survey’, with the objective of capturing change over time. The Chief Medical Officer (CMO) previously identified challenges associated with each, e.g. if the denominator were to include trials already undertaken, how to best capture quality, how these activities compare against other pharmaceutical companies, and whether the percentage of recommendations adopted captures the extent to which the recommendations are appropriate in a trial context.

(Corporate social responsibility meeting 09/22)

Capturing the impacts of patient engagement was deemed ‘challenging’, not least because measures would likely demonstrate the gap between the needs of industry and patient partners (Esmail et al. 2015). However, there was no discussion around capturing the value of the *dialogue* (as discussed above in relation to patient‐made recommendations). Thus, the motives for patient recommendations or the value of the process of engagement (Levitan et al. 2018) and possible qualitative methods to capture them were overlooked. Rather, the inclusion of patients in the CSR model was described as a means to leverage transformation of practice:

[Patient Engagement] should be increased… we were there always to bridge between clinicians and industry, science; we forgot to bridge with patients who are ultimately the ones that should benefit from the provided solutions, who have the basic health or consultation questions, and those should be answered. It turns out that even the advanced therapies that we provide, we are still solving science and symptoms, but not all science and symptoms so there is a residue in what patients experience and what we clinically may call remission may not be true remission from the patient’s perspective… Talking about science is talking about a different level or different perspective than what the patient perspective is; the patient has never been involved for decades in the discussion.

(S4 interview 11/22)

#### 3.1.4 Return on Investment

The need to demonstrate the return on investment of engaging patients in the drug development life cycle was typically discussed by the Patient Engagement Functional Team in terms of proving the monetary value of engagement as the business was actively seeking new global partners, rather than being premised upon translational justice:

When discussing the learning from conducting the pilot patient engagement exercise, the Patient Engagement Functional Team noted that other initiatives were being prioritised within the company, on which S3 reflected: ‘We need a tool to translate what we capture into money. To reduce costs and increase profit. We are a business. We need to put drugs in the market sooner.’

(Team meeting 06/22)

However, exponents of patient engagement expressed a deeper set of motives, while acknowledging that the moral imperative had less traction beyond established PE enthusiasts:

One of the main drivers could be, of course, from a financial perspective, the return on investment, which is super difficult to quantify… There's another one, which is the moral and ethical one. That it’s just the right thing to do. And there's another one that we're trying to leverage right now…which is the cost opportunity, meaning, what are we missing if we don't do it? And sometimes this speaks to people more. So, I'm using this to get more buy‐in, basically.

(S5 interview 08/22)

With the roll‐out of the transformation, S1 and S5 met with a management team to elicit their ‘buy‐in’. Their introductory presentation cited the PFMD framework, introducing engagement in terms of its financial value to the organisation (citing both Getz 2015; Levitan et al. 2018), rather than as an equity‐driven corrective to the absence of patients in the basic sciences (Allyse et al. 2025). It had been presumed that buy‐in would be achieved by asking managers to identify priorities for patient engagement; however, perceived extra work informed resistance:

S6: I can do support, but this is an investment of my time and expertise [laughs] Time is money, eh? …

S7: ‘You have spent a lot of time on this already, but there is a risk that you will over do it!’ You will somehow, sometime lose people, because there is too much. That is what we see happening… [you] have to be careful not to add additional work to people. In the last year you have been able to raise awareness. Everyone is aware, and [staff] are not fully aligned. First, you need to evaluate what is already being done, and what is not well understood. People have ideas, but I am struggling with adding initiatives in the company, it’s a bit conflicting, and I’m with you on patient engagement.

S8: I’m pro‐PE, and we are already doing a lot more than I have ever seen in another company. So, it’s great, and so much work on this, but there’s difference: you’re the expert and we have other priorities, and they are different! Asking everyone for their priorities and being involved, is intense and a difficult question to answer.

S7: You’ve had the opposite effect: ‘Making [patient engagement] process heavy scares people off.

(Management team meeting 10/22)

Thus, a commitment to the ethos of patient engagement was overshadowed by perceived opportunity costs to other organisational functions. Resistance also came from staff who perceived that the process of patient engagement, when aligned with an equality, diversity and inclusivity agenda regarding participant inclusion criteria, could potentially compromise the demonstrable effect size of the trial and thus be untenable:

S9 noted that people with co‐morbidities were not included in the trial, because they are confounders in the statistical analysis. The biostatistician replied that including ‘all of these minorities’ would also skew the trial results (e.g. by reducing the effect size), to the extent that the trial would then become ‘unrepresentative’

(Diversity in clinical trials meeting 12/23)

Although these motives for patient engagement—regulatory requirements, corporate social responsibility and return on investment—were actively used by the Patient Engagement Functional Team to justify their conduct and leverage future intentions, at times these came into direct conflict with the lean corporate business model, deemed to be essential for future financial investment.

### 3.2 Tabooed Motives

The motives of other Biotech ‘functions’, beyond the Patient Engagement Functional Team (e.g., professional specialities or specific departments, and leaders), and those of the patient representatives who were strategically ‘engaged’, further challenged the adoption of formal and standardised models of patient intelligence gathering. Some engagement contexts were off limits for JF’s observation (e.g., strategic meetings between functional teams and patient organisations, where conflict might arise), but also, rather than patient engagement being the good news story for the organisation as intended, as JF was completing fieldwork, staff from the patient engagement team were being released from employment. Thus, the narrative of the patient engagement team was erased or rewritten by peers, patients and managers, which Gerth and Mills (1953) perceive as being tabooed, by which we mean sequestered because the legitimacy of their claim is restricted (by other functional team members), contested (by patient representatives) or repressed (by managers).

#### 3.2.1 Dynamics of Drug Development

The discontinuation of a drug in which patient engagement was being championed further compromised the embedding of formal patient engagement practices into the organisation's workflow. This was seen as potentially creating a vicious cycle of disengagement, rather than the virtuous circle of patient engagement implementation that had been anticipated:

[A] lot of our activities, if not all of ours, are very sensitive to the price on the stock market… [in contrast] the more people working on projects with patients, the more you spread the voice, the more you see things happening, the more positive results come in. And it's a bit of a circle, right? So that generates good things…you have concrete proof points. And you have people buy‐in, and you have concrete experiences, and you speak to people. So, it’s a bit of a movement that you are developing

(S5 interview 08/22)

Initial workshops had forecast that by Year 1 (2023), proof of concept for the value of patient engagement would have been demonstrated and its systematic normalisation commenced. However, as the drug pipeline changed, so did the disease‐specific patient organisation representatives required to input into the process. This meant that patient engagement activities returned to the front end of the pipeline (as new patient contacts were orientated to the drug development and associated patient engagement processes) without having had the opportunity to demonstrate the return on *engagement* later in the drug development life cycle:

S1 suggested revisiting the commitment to patients: Not just invite patients, but do true engagement and true collaboration, via the Patient Engagement Strategy, a six‐year framework with a plan for every year, stating ‘there is a lot to do but we have four years’

(Team meeting 02/24)

For the Patient Engagement Functional Team, this was seen as an opportunity to start again and do ‘true’ patient engagement (e.g., apply learning); however, the associated loss of return on investment meant that processes of engagement needed to become leaner, meaning that discussions of possible equality, diversity and inclusivity (EDI) initiatives—in line with those being developed by regulators (e.g., ICH E8 (R1), which came into effect in 2022, requiring trial sponsors to justify nonrepresentative samples)—were put on hold. Thus, a positive spin on restarting with new patient representatives was that the process could be improved, although the actual consequence was that enactment of the process of learning together did not happen.

Subsequently, instead of employing a CRO with experience of patient engagement, the Patient Engagement Functional Team directly reached out to and engaged a PO, which they had not worked with before. However, in their subsequent patient intelligence exercise, the emphasis was on expediency and coverage of geographical regions, rather than on the patients who had the relevant clinical condition:

In the initial meeting between the Patient Engagement Functional Team and the PO, the PO offered to translate the protocol and questions into a survey rather than conduct focus groups, noting that patient volunteers would not be remunerated for their time (Initial meeting with PO 10/22). At the follow up meeting the PO reported that of the 34 participants who completed the survey, only 20% had a medical condition, while 2% had the *specific* medical condition that was to be the subject of the clinical trial. Both the functional team and PO agreed that this was sufficient insight to inform clinical trial design and conduct

(Follow‐up meeting with PO 3/23)

Here, then, efficiency of data collection was prioritised, along with engagement of future markets, over diversity of disease experience. This ensured that the limitation of disease variance did not hamper the ‘clinical trial science’ (as feared above), but inhibited understanding of the needs of a wider group of patients who would be expected to participate in the trial and ultimately those potentially prescribed the drug if it were then licensed.

#### 3.2.2 Limited Critical Space in Which Patient Engagement Can Occur

Although JF was seconded to Biotech, some research spaces were deemed too sensitive for *formal* observation, most notably the first meeting between the Patient Engagement Functional Team and clinical staff who initiate the journey to drug approval and the first meeting of the Patient Steering Group (PSG). The concept of the PSG was explained as being a consultative body and knowledge exchange platform, with key success factors identified as industry‐facing (e.g., management team endorsement), with none explicitly patient‐facing (PSG meeting 6/22). However, by the second meeting of the PSG (with the patient engagement transformation project on hold due to challenges with the drug pipeline and subsequent organisational restructuring for cost‐effectiveness), patient representatives were asking for more than the current EUPATI‐informed patient engagement offering, specifically in relation to changes to the pipeline and any associated risks to the integrity of patient engagement:

P2: A pitfall is that lots of companies are now doing “patient engagement”. Many people are now doing it, e.g. in marketing. It is unclear, but it also puts the definition at risk. It must remain the *real* meaning of patient engagement….

P3: What is the value obtained from patient engagement, where is it going to lead? How is it going to be evaluated?…

P4: How much were patients involved in devising this [Biotech’ organisational restructuring] strategy?… Please don't make it about, what a number of companies do is: calling patients when the protocol is written. It needs to be so much more. That’s the direction that I’d like to see this go in.

P3: Adding to that point: the end is not the right point you need co‐evaluation as well. You need to think about what meaningful patient engagement is so that it is embedded and doesn't become a casualty in further volatility, for example: it needs to be imbedded cross‐functionally, each group needs a champion to ensure continuity of patient engagement to survive change in personnel….

P4: if you don’t involve patients, things will be more difficult than they need be, you won’t have made the argument to the end user community that what you are doing is appropriate and proportionate and meeting their real needs.

P2: In terms of the business case here, it’s about value: if you want better patient outcomes, you want better patient involvement tools, but how do you know if they work. Talking about your [number of] activities, how many of those were evaluated? What criteria were used? Can we [PSG] help develop tools for patient involvement, so that we can move closer to naming the value here.

(Second PSG meeting 01/23)

The PSG (many of whom were expert patients with many years of experience of working with various pharmaceutical entities) not only wanted to be co‐evaluators of the transformation process but also engaged in the wider business strategy and the identification of metrics to assess both an underpinning theory of change and its outputs. This level of challenge could be due to the patient engagement process enabling transparency and honesty, or it could be down to the experiential knowledge of these patient representatives, and that they were confident in bringing their own motives to the fore. The PSG was viewed as a council of ‘experts’, but the lack of engagement with a wider demographic of patients (e.g., naive or nonexpert patients who would be candidates for clinical trials and new therapies) and healthcare professionals was held to result from outdated regulations:

There are limitations to how we as industry can talk to patients and even health professionals. We should create ways of talking to all those stakeholders with the objective in the end of creating a commonly supported and all involved project each time that we start a collaboration. So, the starting point should be ‘What do want to achieve? What is the level of contribution?’ and then execute… to me that should be [unregulated], and maybe you require a discussion with compliance, health authorities, and committees on what we allow in common agreement… but to me a lot of those limitations are overdone and not relevant or practical. When created for the best purpose, we are still shooting ourselves in the foot. It’s not helping us.

(S4 interview 11/22)

Thus, although members of the Patient Engagement Functional Team were keen to be more inclusive, it was concern for breaching regulatory rules around direct marketing to patients and professionals, rather than the high‐risk business of developing new technologies that would be ‘first in class’ or ‘best in class’, that was perceived as inhibiting collaboration with a wider group of stakeholders.

#### 3.2.3 Patient Engagement as ‘a New Science’

Throughout the transformation project, there was an underlying narrative of engagement as something new, illustrated by an early conversation within the Patient Engagement Functional Team around the process steps required:

S10: When we have the [Patient Voice] manual, we will have all of the processes understood by everyone who is involved…

S11: What are the steps for a patient‐centric trial?

S3: It will depend on every indication and trial. They don’t know, from upfront they start from a scientific assessment, and then they will need to be convinced to adapt their design.

(Team meeting 09/22)

The subsequent iteration of the patient voice manual outlined the status of patient engagement as a work in progress:

The ultimate purpose of the Site Voice process at [Biotech] is to support the development of feasible protocols by proactively addressing the needs of patients and sites… the Patient Voice process is a newly introduced concept within [Biotech] in a continuously evolving environment. This manual should be considered as a living document, which will be regularly updated according to new insights and approaches.

(Patient voice manual 08/23)

Following a further strategic realignment, it was acknowledged that the patient engagement process still required validation with patients to underpin the transformation process (cross‐team workshop 12/23).

One proposition was that validation of the patient engagement process could be undertaken with EUPATI Fellows, rather than POs, to expedite the validation process. However, in a subsequent discussion of ‘the new science’, patient engagement seemed to be conflated with data‐driven precision medicine, thus further emphasising tensions between the different motives and objectives of patient engagement:

P5: when you see that patient engagement is becoming a science…What’s your perspective on how this patient involvement will evolve and the methodology of patient engagement that is becoming more and more scientific?

S12: For me, if you say scientific… it’s going to be very practically needed…could it be electronic patient records…So, I get real‐time outcome measurement system, which can be done on a personal basis. So you can go into that database with your data, and ask: what is the best option for me, at my time? Where even can I find a clinical trial that fits best to me. And that’s part of their business model. Can we direct you to the right trial for, in this network?

(PSG meeting 06/24)

Thus, the science of patient engagement was seen as transactional and extractive, rather than relational and inclusive.

## 4 Discussion

This research provided a novel example of knowledge exchange between clinicians, patients and industry. Through conversations with patient advisors and clinical partners, we drew on established research about how vocabularies of motive both inscribe and manifest social practice and actions (Mills 1940). By doing so, we identified a range of motives to enable and inhibit meaningful widening patient engagement in drug development.

Members of the Patient Engagement Functional Team drew upon externally established or ‘premium’ motives, in the form of standardised industry‐sponsored frameworks, in an attempt to leverage organisational support for patient intelligence structures to inform drug development. At the initiation of the transformation process, credibility was sought through the employment of external industry consultants who delivered a recognised business strategy to enable development of a patient intelligence process. Subsequently, expert‐led frameworks, such as EUPATI, were drawn on because, for S1, ‘it is a published reference…important to people that we work with who are scientific minds’ (PSG meeting 01/23). This is in keeping with Gerth and Mills' (1953) assertion that individuals attempt to control a dynamic situation in which they are seeking the establishment of a new norm by invoking a premium motive to manipulate or influence current business practice. However, the constant business reconfiguration ensured that there was never enough time to establish the work but, paradoxically, plenty of time to start again (Webb 1996). Thus, patient engagement was framed by the wider organisation as a function of, rather than contributor to, business stability, which meant that opportunities for the Patient Engagement Functional Team to demonstrate their return on investment in €, and ultimately add value to the organisation, went unrealised.

In the face of constant organisational reconfiguration, the call to *true engagement* and *true collaboration* resonates with recent calls to reimagine the language of patient engagement to avoid the rhetoric of inclusion and to foreground the power dynamics required to tackle inequalities (Reed et al. 2024). To that end, we saw how the Patient Engagement Functional Team sought to disrupt the current organisational status quo by exploiting the framing of patient engagement within the current EU CSR initiative as a site to situate actions that address societal concerns about industry priorities (Sykes and Matza 1957). However, the managerial sanctioning of the patient engagement initiative, in terms of its measurement with existing key performance indicators, mitigated the perceived risk associated with engaging with (potentially limitless) patient desires (Suddaby and Greenwood 2005). Limited understanding of qualitative methodology and its epistemic potential, more generally (Frost et al. 2023), neutralised the legitimacy of the Patient Engagement Functional Team's attempts to transform existing power relations through their action (Sykes and Matza 1957). For example, the observed practices of surveying patient representatives about their satisfaction with the engagement process are at odds with demonstrating if their input is ethical, effective or comparable (Robert et al. 2018). Qualitative enquiries into the motives of both PSG members and patients who participate in patient intelligence as ‘subjects’ could yield deeper understanding of how the processes of engagement are perceived by patient partners (Phoenix et al. 2018) when trials are successful but also unsuccessful (Frost et al. 2025a; Frost et al. 2025b).

We identified several instances where disclaimers, or negative typifications of patient engagement (Hewitt and Stokes 1975), were employed by the members of other functional teams, who perceived the patient engagement project as disrupting the typical flow of clinical trial science, for example, by initiating a patient engagement strategy that was ‘process heavy’ or at odds with the need for a homogeneous trial population to demonstrate a statistical effect. This meant that attempts by the Patient Engagement Functional Team to establish regular dialogue with other functional teams were repressed so as not to interfere with the established drug development process. This finding resonates with those of other researchers, who are divided as to whether the drive for trial efficiency is possible at the same time as widening patient engagement to incorporate groups currently marginalised by, or excluded from, the drug development process (Zvonareva 2023; Egher and Zvonareva 2025). There is evidence of growing social purpose and tools available to expand the demographic of patients involved in clinical trials (e.g., as ‘subjects’) (Venkatakrishnan and Benincosa 2022). However, in terms of engagement in drug design, currently industry‐defined practices ensure that patient advocates from the Global North are over‐represented in drug development research, whereas only those who have substantial knowledge of drug development are ‘engaged’ (Zvonareva et al. 2022; Egher and Zvonareva 2023; Zvonareva 2023). In this example, attempts by the Patient Engagement Functional Team to demonstrate a return on engagement were deemed unworkable or taboo against an established premium motive, for example, the identification and licensing of a new molecule and subsequent financial return on investment.

We also observed how PO representatives who had established relationships with the Patient Engagement Functional Team used disclaimers of the current patient engagement offer to petition for a greater role in setting and enacting the patient engagement agenda within Biotech. Mills proposed that the exploration of motives could enable sociologists to identify the general conditions, character, mechanisms and typification of social actions (Mills 1940). Here we have shown that, where Gerth and Mills (1953, 123) contend ‘pecuniary motives tend to form a sort of common denominator’, other vocabularies are treated as ‘shams’ or ‘facades’; similarly, the constant techno‐economic flux of a small biotechnology company situated patient engagement as transactional and ‘nice to have’ (S4). We contend that any framework aimed at addressing the epistemological motives central to the historical foundations for establishing patients as partners must be premised on a relational ontology that seeks to redistribute power beyond the enclosure of current pharmaceutical knowledge (Geiger and Kleinhout‐Vliek 2026). We also see vocabularies of motive as a potential framework for capturing and explaining co‐created intentions: This could be an equity‐based approach that prioritises sustained commitment to patient partners—beyond a single project with patients involved in the identification and evaluations that are important to them (Sayani and Maybee 2025); or a more radical commitment to translational justice that seeks to dismantle not only the current pharmaceutical discourse but also the fiscal and regulatory structures which currently circumscribe which patient voices are permissible and on whose terms (Allyse et al. 2025). This would enable industry to meet their moral obligation to a wider demographic of patients by foregrounding their sustainable engagement throughout all stages of both the drug and business development life cycles and establishing a more equitable vocabulary of motives.

Where previous sociologists have employed vocabularies of motive in an exploratory sense, we have employed them as an explanatory tool and, in an industrial context, to examine not only which actors are successful, or not, in their attempts to situate actions to enact their knowledge claims but also to identify the impact of that enacting on what is purported to be a shared goal (here, patient engagement) (Suddaby and Greenwood 2005). In doing so, we have demonstrated the potential of vocabularies of motive as an evaluative tool to identify which vocabularies prevail in a given context and which vocabularies are constrained or discounted. This matters because, as Mills suggested, shifting the vocabularies of motive to one that is acceptable is a mechanism of societal control (1940). Thus, we see scope in creating typical frames of ‘socially situated clusters of motive’ (1940, 913) to better extend our understanding of what enables meaningful patient engagement in the development of drugs, but also, as an explanatory tool more generally, for sociologists to explore the extent to which the social order is circumscribed and potential sites for new shared motives and situated actions.

A limitation of this work is that access to industry meetings, practices and documents was controlled by the industry host (S1), which may give a partial view of industry partners, for example, those who may have been enthusiasts for patient engagement (Crowhurst and Kennedy‐Macfoy 2013). Despite this, this paper offers learning from a knowledge transfer partnership between industry and academia which sought to identify how a wider demographic of patients may be enabled to participate in both clinical trials and trial design. As such, we offer vocabularies of motive as both a meaningful framework for future practice and as a conceptual lens through which to assess whose interest an organisation’s patient engagement strategy may or may not serve.

## 5 Conclusion

This theoretically informed investigation of drug development has identified the challenge of identifying and operationalising the mechanisms required for optimal patient engagement in drug development. Of note is the absence of clearly delineated patient‐focused vocabularies for capturing processes and outcomes that reflect the many motives for undertaking patient engagement, specifically those relevant to the patients who are engaged by virtue of belonging to patient organisations but also patients outside of those organisations, whose voices are currently under‐represented in the drug development life cycle. This analysis has demonstrated tensions between capturing notifiable impacts and capturing the process associated with ‘being engaged’, as well as tensions between initiatives to bring drugs to market faster and those to include often ignored populations, which may require more resources to enable engagement. As such, the observed vocabularies of motive employed by the Patient Engagement Functional Team in Biotech and the patient representatives from patient organisations can be perceived as having been inhibited by the wider vocabularies employed by the Biotech managers and other functional teams. The prioritisation of other business agendas negated the establishment of a patient‐driven collaborative patient engagement process, as well as the potential for a wider demographic of patients to gain a voice in drug trial design and conduct. This research also provides an example of how collaborations between academia, patients and industry can foster shared learning to advance patient engagement in the drug development life cycle, but also the health research landscape more generally. It also describes and explains how vocabularies of motive are valuable for the academic field more generally.

## Author Contributions

**Julia Frost:** conceptualization, investigation, funding acquisition, methodology, software, writing – original draft, formal analysis, project administration, resources, data curation. **Widening Engagement Patient Advisory Group:** validation, supervision, investigation, funding acquisition, writing – review and editing. **Jessica Mandizha:** conceptualization, funding acquisition, writing – review and editing, validation, supervision. **Chantal van den Dungen:** validation, supervision, data curation, writing – review and editing. **Catherine Pope:** conceptualization, investigation, funding acquisition, writing – review and editing, methodology, data curation, supervision, formal analysis, validation.

## Funding

This research was funded by the Medical Research Council (Grant MR/W003732/1).

## Ethics Statement

Ethical approval was provided by London—Riverside Research Ethics Committee (REC reference: 22/LO/0186; IRAS project ID: 304904).

## Consent

All participants gave their written consent for the interview in which they participated to be recorded and declared that they understood that the interview would be typed up (transcribed), with all of the information anonymised. For observations, a privacy notice was shown prior to all meetings that the industry host identified as acceptable for formal observations, which, in keeping with company policy, stated that recordings would be solely used for the data analysis and anonymised extracts of data may be published and used in further research.

All participants gave their written consent for the interview in which they participated to be used in publications, with all of the information anonymised. For observations, a privacy notice was shown prior to all meetings that the industry host identified as acceptable for formal observations, which, in keeping with company policy, stated that recordings would be solely used for the data analysis and anonymised extracts of data may be published and used in further research.

## Conflicts of Interest

The authors declare no conflicts of interest.

## Data Availability

Transcripts and field notes will not be shared in their entirety to protect the anonymity of study participants. However, requests for excerpts of the data will be considered on an individual basis. Please contact the corresponding author.
